# Influence of early fluid overload on bronchopulmonary dysplasia in very low-birth-weight infants

**DOI:** 10.3389/fped.2022.980179

**Published:** 2022-10-11

**Authors:** Yu-Jie Li, Xiao-Fang Zhu, Jian-Hong Liu, Xiao-Qian Yi, Hao He

**Affiliations:** Department of Neonatology, Jingzhou Hospital Affiliated to Yangtze University, Jingzhou, Hubei, China

**Keywords:** bronchopulmonary dysplasia, very low-birth-weight infant, preterm infant, risk factor, fluid overload

## Abstract

**Objective:**

This study aimed to determine the influence of fluid overload on bronchopulmonary dysplasia (BPD) in very low-birth-weight infants (VLBWI) within 1 week after birth.

**Methods:**

This was a retrospective case control study conducted in the Jingzhou Central Hospital. The clinical data of VLBWI (with a birth weight [BW] < 1,500 g and 26 weeks ≤ gestational age [GA] < 32 weeks) who were admitted to the neonatal intensive care unit of this hospital from January 2016 to December 2021 were analyzed retrospectively. A total of 157 cases were enrolled and divided into a BPD group (*n* = 60) and a non-BPD group (*n* = 97) according to whether BPD was present. The general condition, fluid intake, and fluid overload of the two groups of neonates within 1 week after birth were compared. The logistic regression was used to assess the association between infant characteristics and BPD. The ROC curve was used to assess how well the 7 day cumulative fluid overload predicted BPD, and to identify an optimal cut off for prediction.

**Results:**

The comparison of the patients' general condition revealed that the neonates in the BPD group had a younger GA, lower BW, lower 5-min Apgar score, longer duration of invasive mechanical ventilation, and higher incidence of intrauterine infections and administration of surfactants (*P* < 0.05). The differences in the other indicators were not statistically significant between the two groups. The logistic regression analysis revealed that a younger GA, the presence of intrauterine infection, and a 7-day cumulative fluid overload were the risk factors for the development of BPD. A ROC curve was plotted with the 7-day cumulative fluid overload as the test variable and BPD as the status variable. The area under the curve was 0.75 (95% confidence interval: 0.664–0.826, *P* = 0.042), with a sensitivity of 76.7% and a specificity of 70.1%, corresponding to a 7-day cumulative fluid overload of 36.2%.

**Conclusion:**

A younger GA, the presence of intrauterine infection, and a 7-day cumulative fluid overload were risk factors for the development of BPD. A 7 day cumulative fluid overload threshold of 36.2% best predicted the development of BPD.

## Introduction

With the advances in neonatal intensive care unit (NICU) care and perinatal technology, the birth and survival rates of very low-birth-weight infants (VLBWI) and extremely low-birth-weight infants (ELBWI) have increased considerably, and the incidence of bronchopulmonary dysplasia (BPD) also increases along with it (1). Currently, BPD remains the most common and serious chronic respiratory disease in preterm infants (2), and in addition to a high mortality rate, survivors often have hyper-reactive airway disease, recurrent lower respiratory tract infections, and growth retardation. The effective prevention and treatment of BPD remain a focus of attention in neonatology.

Abnormal postnatal fluid status may promote the development of BPD. The 1st week after birth is a critical period for intrauterine to extrauterine transition, and although the relevant guidelines provide recommendations for fluid management, infants are prone to developing occult fluid overload (FO) due to differences in healthcare centers and units, individual patients, and diseases. Fluid overload may lead to reduced pulmonary compliance and an increased need for oxygen and mechanical ventilation (MV) (3), while MV-related lung injury and ventilator-associated pneumonia predispose neonates to develop BPD (2).

Previous studies (4, 5) found that a high fluid intake in the early postnatal period was associated with the occurrence and severity of BPD in VLBWI. A recent study (6) investigated the association between fluid and sodium status in the first 10 postnatal days and death/ BPD among infants born, they concluded that a higher CFB in the first 10 days after delivery was associated with higher odds of death/BPD among preterm infants. It has also been reported that there is an association between total sodium intake and BPD within the first 2 weeks of life among ELBW infants (7). However, no study has identified an association between early fluid overload and BPD in ELBWI. In the present study, we determined the influence of fluid overload on bronchopulmonary dysplasia (BPD) in very low-birth-weight infants (VLBWI) within 1 week after birth. The clinical data of VLBWI who were admitted to the NICU of Jingzhou Central Hospital from January 2016 to December 2021 were collected retrospectively to observe and analyze the correlation between early fluid overload and BPD and to determine whether 3 and 7-day cumulative fluid overloads are risk factors for BPD.

## Materials and methods

### Study design and patients selection

This was a retrospective case control study, following the STROBE guidelines (8). The study population was newborns admitted to the NICU of Jingzhou Central Hospital at the Second Clinical College of Changjiang University between January 2016 and December 2021 who met the following inclusion criteria and did not meet the exclusion criteria.

The inclusion criteria were the following concomitant conditions: (1) neonates who were born in our obstetrics department, (2) newborns admitted to our NICU within 24 h after birth, (3) neonates with a birth weight (BW) < 1,500 g, (4) 26 weeks ≤ GA < 32 weeks, and (5) newborns with a hospital stay of ≥14 days.

Those who met at least one of the following exclusion criteria were excluded from the study: (1) newborns with severe asphyxia, (2) newborns with inherited metabolic diseases or severe congenital malformations, (3) newborns with severe congenital cardiovascular diseases, (4) newborns with gastrointestinal perforation that was not caused by neonatal necrotizing enterocolitis (NEC) and required surgery, and (5) newborns with incomplete data. The present study was approved by the ethics committee of the hospital.

### Grouping and fundamental therapy

The enrolled newborns were divided into two groups based on the presence of BPD. After admission, all the newborns were treated with respiratory support, enteral and parenteral nutrition, maintenance of vital signs, stabilization of the internal environment, and symptomatic support. Neonates with hemodynamic abnormalities caused by patent ductus arteriosus (hs PDA) were treated with medications, and no special treatments were administered.

For this study, the diagnosis of BPD was based on the diagnostic criteria of NICHD in 2001. BPD was defined as death due to pulmonary parenchymal disease and respiratory failure with the exclusion of NEC, severe intraventricular hemorrhage (IVH), sepsis between 14 days of postnatal age and 36 weeks of adjusted GA, and those in whom oxygen inhalation was still required 28 days after birth (9, 10). In this study, Oxygen was still needed at 28 days after birth. However, we found that some newborns died before the hospital stay reached 28 days.

### Data collection

With the use of the hospital information system database in our hospital, the patients' general information (gender, time of admission, mode of delivery, GA, BW, 5-min Apgar score, multifetal pregnancy administration of surfactants and antibiotics after birth, duration of invasive MV, etc.), daily fluid intake (including intravenous fluids, blood products, medications and flushes, and enteral nutrition) and output (stool volume, urine volume, and gastrointestinal drainage), daily weight, and adverse outcomes (BPD, PDA, late-onset sepsis [LOS], NEC, grade III–IV IVH, and pulmonary hemorrhage [PH]) were collected.

### Daily fluid overload calculation method

Generally, the daily fluid overload is calculated using the following method (11):


$$\begin{array}{l} Daily\;fluid\;overload\;\left(\%\right)=\left(fluid\;intake-fluid\;output\right)\;\left(L\right)\\ \;\times 100\%/BW\;\left(kg\right) \end{array}$$

However, considering BW differences, the formula used in the present study was as follows:


$$\begin{array}{l} Daily\;fluid\;overload\;\left(\%\right)=\left(fluid\;intake-fluid\;output\right)\;\left(L\right)\\ \;\times 100\%/Daily\;weight\;\left(kg\right) \end{array}$$

The daily fluid overload, 3-day cumulative fluid overload, and 7-day cumulative fluid overload were calculated separately to reflect the early fluid overload.

### Nursing standards and definitions

During the study period, the nursing standard included an initial total liquid intake of 80–100 mL/kg/D, followed by an increase of 20 mL/kg/D per day. No sodium was added to the parenteral nutrition supplemented in the initial 72 h. The sodium intake is usually supplemented with the physiological requirement of 3 mmol/kg/day from the 4th day and adjusted according to the blood sodium concentration. The infants were placed in an incubator, and the relative humidity was generally 70% in the first 7 days and 50% later. The nurse weighed the baby with the same scale at 8:00 every morning, and counted the liquid intake and output of the previous day.

PDA was diagnosed by echocardiography; NEC was diagnosed according to modified Bell stage by combining clinical manifestation and imaging examination (12); Grade III–IV intracranial hemorrhage was diagnosed according to the graduation criteria proposed by papile (13) (Grade III: intraventricular hemorrhage with ventricular dilatation; Grade IV: intraventricular hemorrhage with extrameningeal hemorrhage).

Diagnostic criteria of LOS: The disease usually starts after 72 h of birth, including clinical diagnosis and a definite diagnosis. The former is one of the following conditions under the premise of clinical abnormal manifestations: blood non-specific examination ≥ 2 positive; abnormal cerebrospinal fluid examination; DNA or antigen of special bacteria were detected in blood. The latter is positive in blood culture or cerebrospinal fluid (or other sterile cavity fluid) culture under the premise of clinical abnormal manifestations.

The diagnostic criteria of PH: sudden dyspnea and/or increased thin moist rale in the lungs; bleeding red liquid at the mouth and nose and/or bloody liquid in the endotracheal tube.

### Statistical analysis

The measurement data were expressed as mean ± standard deviation (*x* ± *s*) or median (*M*) (min–max). Countable data were expressed as the number of cases (*n*) (%), and the Chi-squared test or Fisher's exact probability method was used for intergroup comparisons. A value of *P* < 0.05 was considered statistically significant. Binary logistic regression was used to explore the relationship between the early fluid overload and BPD, and the receiver operator characteristic (ROC) curve was used to determine the optimal threshold.

## Results

**Figure 1:**
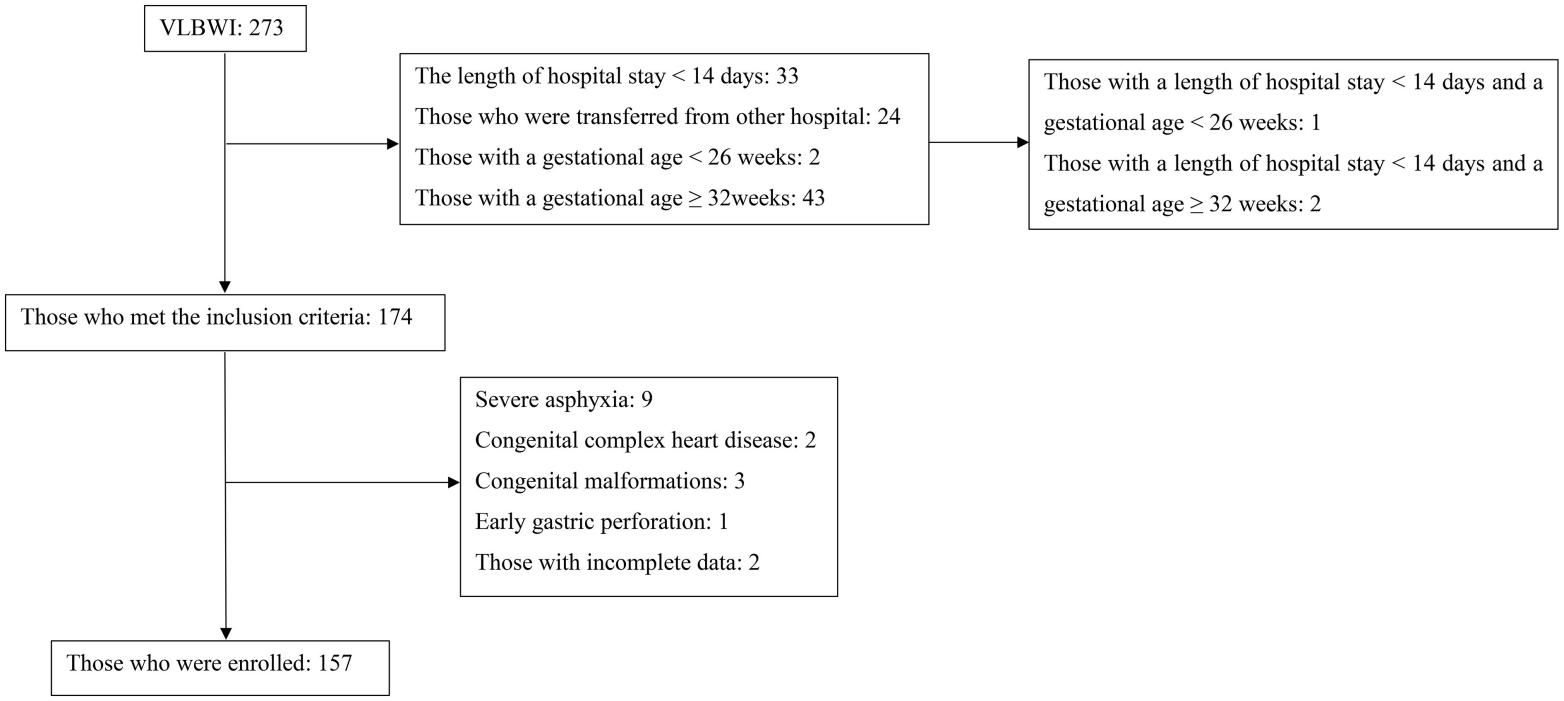
The study population.

A total of 273 VLBWI were admitted to the NICU during the study period. Of these, 174 met the inclusion criteria; after the exclusion of 17 cases, 157 neonates were finally enrolled in the present study (as illustrated in Figure 1).

With the comparison of the general characteristics between the two groups of VLBWI, it was found that, compared with those in the non-BPD group, the neonates in the BPD group had a younger GA, lower BW, lower 5-min Apgar score, longer duration of invasive MV, and higher incidence of intrauterine infections and administration of surfactants after birth (see Table 1).

**Table 1:** Comparison of the general characteristics between the non-BPD group and the BPD group in VLBWI.

	**The non-BPD group (97)**	**The BPD group (60)**	***P***
Male [n (%)]	45 (46.4%)	37 (61.7%)	0.063
Cesarean delivery [n (%)]	33 (34.0%)	22 (36.7%)	0.736
Gestational age [M (min~max), week]	30.2 (27.1~31.9)	29.1 (26.7~31.7)	<0.001^*^
The age at admission [*M* (Min~Max), minute]	10 (8~60)	10 (10~60)	0.990
Birth weight [*M* (min~max), g]	1320 (1020~1490)	1215 (790~1490)	<0.001^*^
5-min Apgar score (mean ± SD, min)	8.89 ± 0.776	8.47 ± 0.453	0.002^*^
Small for gestational age	2 (2.1%)	1 (1.7%)	1^a^
Gestational diabetes mellitus [n (%)]	7 (7.2%)	7 (11.7%)	0.342
Gestational hypertension / Early stage of eclampsia / preeclampsia [n (%)]	22 (22.7%)	8 (13.3%)	0.148
Intrauterine infection [n (%)]	24 (24.7%)	33 (55.0%)	<0.001^*^
Antenatal corticosteroids administration [n (%)]	55 (56.7%)	41 (68.3%)	0.146
Multifetal pregnancy [n (%)]	34 (35.1%)	23 (38.3%)	0.678
Placental abruption [n (%)]	2 (2.1%)	2 (3.3%)	0.637^a^
Premature rupture of membranes [n (%)]	27 (27.8%)	12 (20.0%)	0.270
Threatened prematurity [n (%)]	5 (5.2%)	1 (1.7%)	0.408^a^
Surfactant [n (%)]	37 (38.1%)	42 (70.0%)	<0.001^*^
Antibiotics during hospitalization [n (%)]	94 (96.9%)	60 (100%)	0.287^a^
MV duration [*M* (min~max), d]	0 (0~11)	0 (0~62)	<0.001^*^

The ^*^symbol indicates the statistically significant values of *P* < 0.05.

a Fisher's exact probability.

The incidences of PDA, NEC, PH, grade III–IV IVH, and LOS in the BPD group were 50.0, 8.3, 28.3, and 3.3%, respectively, which were higher than that in the non-BPD group (37.1, 2.1, 16.5, and 0% respectively). Besides, the differences were not statistically significant (*P* > 0.05) (see Table 2).

**Table 2:** Comparison of the incidences of complications between the BPD group and the non-BPD group in VLBWI.

	**The non-BPD group (97)**	**The BPD group (60)**	***P***
PDA [*n* (%)]	36 (37.1%)	30 (50.0%)	0.112
NEC [*n* (%)]	2 (2.1%)	5 (8.3%)	0.107^a^
LOS [*n* (%)]	16 (16.5%)	17 (28.3%)	0.077
Grade III–IV IVH [*n* (%)]	2 (2.1%)	1 (1.7%)	1^a^
PH [*n* (%)]	0 (0)	2 (3.3%)	0.145^a^

a Fisher's exact probability.

The daily fluid intake in the BPD group was higher than in the non-BPD group from days 1 to 5 after birth (*P* < 0.05). The daily fluid intake from days 6 to 7 after birth was higher in the BPD group than in the non-BPD group, and the differences were not statistically significant (*P* > 0.05) (see Table 3). From the results, it also showed that the fluid intake was increasing from the day 1 to day 7 both in the non-BPD group and BPD group. The fluid intake of non-BPD group was 161.5 ± 20.5 mL/kg/d on day 7, and 165.6 ± 24.6 mL/kg/d of BPD group on day 7.

**Table 3:** Comparison of the fluid intake and overload within 7 days between the two groups of VLBWI.

		**The non-BPD group (97)**	**The BPD group (60)**	***P***
Fluid intake (mL/kg/d)	The fluid intake on the 1st day [*M* (min–max), ml/Kg/d]	58.7 (21.7–149.2)	68.6 (26.7–180.8)	0.009^*^
	The fluid intake on the 2nd day [*M* (min–max), ml/Kg/d]	99.1 (61.5–160.2)	116.3 (66.4–196.5)	<0.001^*^
	The fluid intake on the 3rd day [*M* (min–max), ml/Kg/d]	125.9 (89.9–186.5)	136.4 (91.2–204.8)	0.010^*^
	The fluid intake on the 4th day [*M* (min–max), ml/Kg/d]	142.2 (94.9–215.2)	153.5 (97.9–221.1)	0.023^*^
	The fluid intake on the 5th day [*M* (min–max), ml/Kg/d]	152.7 (111.4–212.3)	157.6 (123.8–227.3)	0.013^*^
	The fluid intake on the 6th day (x ± s, ml/Kg/d)	157.7 ± 20.7	163.5 ± 25.1	0.117
	The fluid intake on the 7th day (x ± s, ml/Kg/d)	161.5 ± 20.5	165.6 ± 24.6	0.266
Fluid overload (mean ± SD)	The fluid overload on the 1st day	2.6 ± 2.3	3.3 ± 2.6	0.129
	The fluid overload on the 2nd day	2.0 ± 2.7	3.3 ± 3.0	0.004^*^
	The fluid overload on the 3rd day	3.6 ± 2.7	4.6 ± 3.0	0.033^*^
	The fluid overload on the 4th day	5.8 ± 2.9	6.9 ± 2.7	0.018^*^
	The fluid overload on the 5th day	6.9 ± 2.7	8.1 ± 3.1	0.009^*^
	The fluid overload on the 6th day	6.1 ± 2.7	8.0 ± 2.9	<0.001^*^
	The fluid overload on the 7th day	6.5 ± 2.5	7.4 ± 3.0	0.047
	The 3-d Cumulative fluid overload	8.2 ± 4.7	11.2 ± 5.5	<0.001^*^
	The 7-d Cumulative fluid overload	33.4 ± 9.0	41.5 ± 9.6	<0.001^*^

* P<0.05 was considered statistically significant.

The daily fluid overloads were higher in the BPD group than in the non-BPD group from days 2 to 7 after birth (*P* < 0.05). The daily fluid overload was higher in the BPD group than in the non-BPD group on day 1 after birth, but the difference was not statistically significant (*P* > 0.05). The 3 and 7-day cumulative fluid overloads (41.5 ± 9.6) were significantly higher in the BPD group than in the non-BPD group (33.4 ± 9.0) (*P* < 0.01) (see Table 3).

A univariate analysis was used to screen the factors correlated with BPD, including GA, BW, 5-min Apgar score, presence of intrauterine infection, use of surfactant, and duration of invasive MV as well as the 3 and 7-day cumulative fluid overloads. A multivariate logistic regression analysis model was constructed. The details was showed in Supplementary Table 1. The results revealed that GA (odds ratio [OR]: 0.525, 95% confidence interval [CI]: 0.336–0.819), intrauterine infection (odds ratio [OR]: 3.242, 95% confidence interval [CI]: 1.328–7.913), and 7-day cumulative fluid overload (odds ratio [OR]: 1.063, 95% confidence interval [CI]: 1.006–1.123) were independently correlated with the occurrence of BPD.

An ROC curve was plotted using the 7-day cumulative fluid overload as the test variable and BPD as the status variable. The area under the curve (AUC) was 0.75 (95% confidence interval [CI]: 0.664–0.826, *P* = 0.042), with a sensitivity of 76.7%, a specificity of 70.1%, and a Youden index of 0.468; these results corresponded to a 7-day cumulative fluid overload of 36.2%.

## Discussion

The founding of this study showed that GA and intrauterine infection were independent risk factors for BPD which was partially consistent with the study by Alonso et al. (14) on 202 VLBWI in which GA, the application of invasive MV within 1 day after birth, and the presence of nosocomial infection were identified as the main risk factors for the development of BPD.

The present study found the incidence of LOS to be higher in the BPD group than in the non-BPD group, but the difference was not statistically significant (*P* > 0.05), as shown in Table 2. This finding might have been related to the small sample size, and further research should be conducted to confirm this finding. Kamali Khan et al. (15) identified intrauterine infection as one of the independent risk factors for BPD in neonates, which was consistent with the results of the present study. Intrauterine infection might affect fetal alveolar development *via* cytokine-mediated inflammatory response (16, 17).

Finally, the present study revealed that neonates in the BPD group had a lower 5-min Apgar score and a higher incidence of surfactant administration after birth. Thus, it is suggested that the development of BPD might be closely correlated with early primary diseases, such as neonatal respiratory distress syndrome, as well as ischemia and hypoxia.

The Table 3 showed that the daily fluid intake of the BPD group was significantly higher than that of the non-BPD group on the 1st to 5th day after birth (*P* < 0.05), and the daily fluid intake of the BPD group was higher than that of the non-BPD group on the 6th to 7th day after birth, but the difference was not statistically significant (*P* > 0.05). This may be due to the fact that most clinicians may be aware of the problem of fluid overload and make appropriate adjustments on days 6-7, especially when no significant physical weight loss is found. This finding is similar to the results of Oh et al., (4) who suggested that a high fluid intake (ml/kg) in the first 10 days after birth in 1,382 ELBWI was correlated with the risk of BPD development. Additionally, Al-Jebawi et al. (5) concluded that a high daily fluid intake (ml/kg) in VLBWI during the 1st week after birth was correlated with the severity of BPD. However, the above two studies were limited to daily fluid intake and ignored output, and neither conducted systematic studies of the daily and cumulative fluid overloads. Accumulated studies have been carried out to specify the relationship of sodium intake and IVH in preterm infants. There is no significant difference of the incidenceof IVH between the sodium-restricted group and the sodium-maintenance group in 17 very VLBW infants, while they don't specify the clear dose of “inadvertent” sodium intake (18). Barnette's study reported that the increased odds ratio of severe IVH associated with an average sodium intake >2.5 mEq/kg/day (19). Another study has also demonstrated that the risk for severe IVH was strongly associated with total sodium intake >3.0 mEq/kg/day or sodium intake from fluids other than transfusions >2.5 mEq/kg/day during the initial 4 days of life (20).

In the present study, the investigation revealed that the daily fluid overloads were higher in the BPD group than in the non-BPD group from days 2 to 7 after birth (*P* < 0.05). The daily fluid overload was higher in the BPD group than in the non-BPD group on day 1 after birth, but the difference was not statistically significant (*P* > 0.05). This might have been due to the inconsistent admission times of the patients on the first day. The 3 and 7-day cumulative fluid overloads were significantly higher in the BPD group than in the non-BPD group (*P* < 0.01), and the increased 7-day cumulative fluid overload was an independent risk factor for the development of BPD (*P* < 0.05, odds ratio: 1.066, 95% CI: 1.061–1.119). In a previous study concerning the fluid overload in neonates, Matsushita et al. (3) found that a 3-day cumulative fluid overload ≥15% was correlated with increased mortality and prolonged MV duration in VLBWI. Rallis et al. (21) concluded that a day-1 fluid overload ≥5% was significantly associated with hs PDA in VLBWI. More recently, Soullan et al. (6) observed the 10-day postnatal fluid status in 191 preterm infants with GA ≤ 28 weeks and concluded that fluid overload in the first 10 days was correlated with the occurrence of a BPD/composite death outcome.

All these results indicate that fluid overload in the 1st week after birth might be of considerable concern to clinicians.

**Figure 2:**
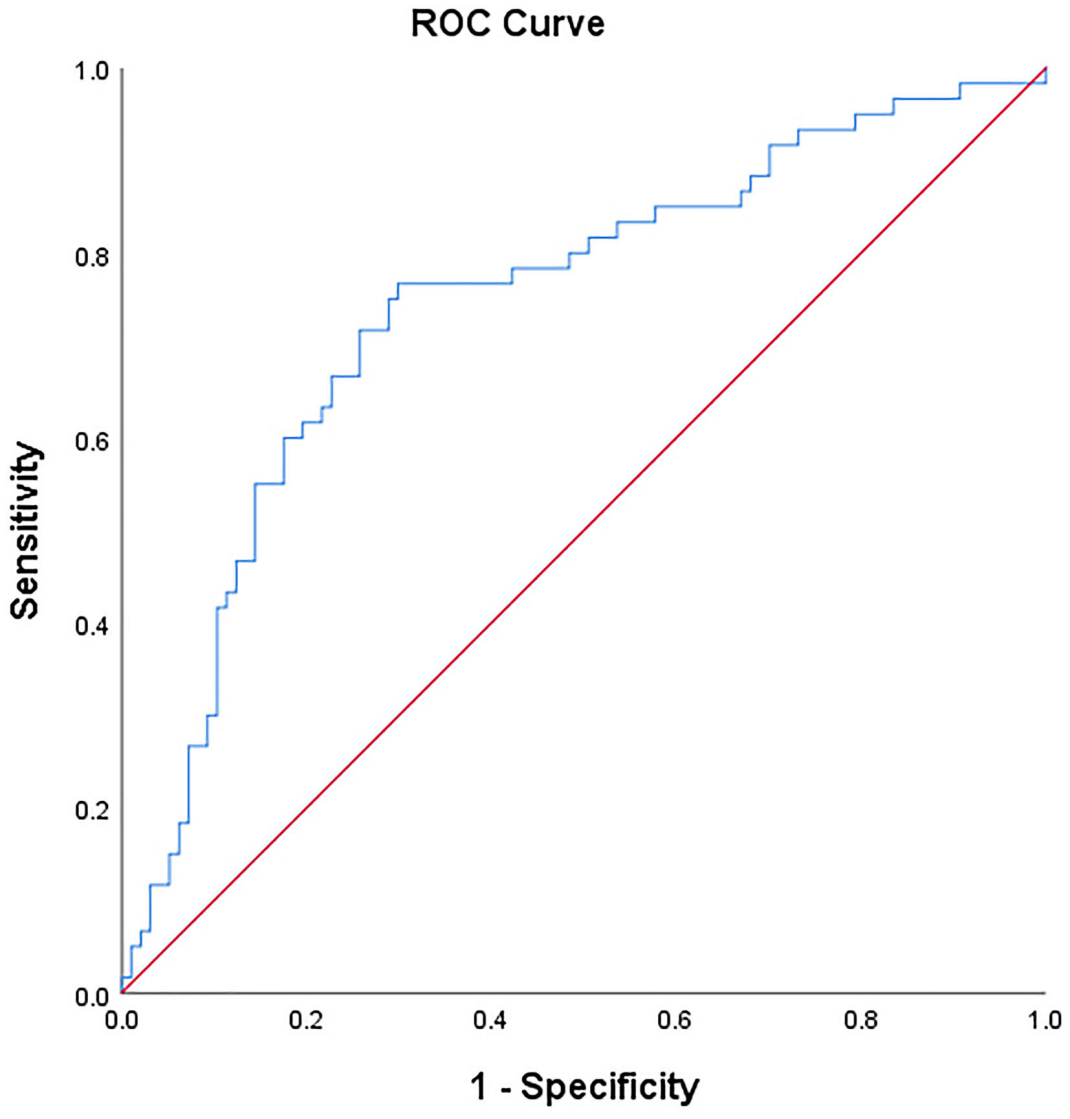
The ROC curve of BPD and the s-cumulative fluid overload.

In the present study, the ROC curve was plotted using the 7-day cumulative fluid overload as the test variable. The results showed that the AUC of the ROC curve was >0.7, and when the maximum Youden index was 0.468, the corresponding optimal threshold of the 7-day cumulative fluid overload was 36.2%, the sensitivity was 76.7%, and the specificity was 70.1%, indicating that the 7-day cumulative fluid overload might have some value in predicting the occurrence of BPD (see Figure 2).

The present study yielded some meaningful results, but there were some limitations: (1) It was a retrospective clinical study spanning 5 years, with some missing data. (2) The small sample size of the VLBWI might have limited the emergence of meaningful results due to the limitations of the inclusion criteria. (3) The relationship between fluid overload and caloric intake was not addressed in the present study.

## Conclusion

In this study, we determined the influence of fluid overload on bronchopulmonary dysplasia (BPD) in very low-birth-weight infants (VLBWI) within 1 week after birth. It was found that a young GA, the presence of intrauterine infection, and the 7-day cumulative fluid overload were the independent risk factors for the development of BPD in ELBWI. A 7-day cumulative fluid overload threshold of 36.2% best predicted the development of BPD among VLBWIs.

## Data availability statement

The original contributions presented in the study are included in the article/Supplementary material, further inquiries can be directed to the corresponding author/s.

## Ethics statement

The study was conducted in accordance with the Declaration of Helsinki (as was revised in 2013). The study was approved by Ethics Committee of the Jingzhou Hospital Affiliated to Yangtze University. The parents of all newborns provided informed consent for their infants' medical records to be used.

## Author contributions

Y-JL and X-FZ: Conception and design of the research. X-QY and HH: Acquisition of data. Y-JL: Analysis and interpretation of the data. Y-JL and J-HL: Statistical analysis. Y-JL: Writing of the manuscript. X-FZ: Critical revision of the manuscript for intellectual content. All authors read and approved the final draft. All authors contributed to the article and approved the submitted version.

## Conflict of interest

The authors declare that the research was conducted in the absence of any commercial or financial relationships that could be construed as a potential conflict of interest.

## Publisher's note

All claims expressed in this article are solely those of the authors and do not necessarily represent those of their affiliated organizations, or those of the publisher, the editors and the reviewers. Any product that may be evaluated in this article, or claim that may be made by its manufacturer, is not guaranteed or endorsed by the publisher.
