# Supplementary material for: Influence of early fluid overload on bronchopulmonary dysplasia in very low-birth-weight infants
Source: Front Pediatr. 2022 Oct 11;10:980179. doi: 10.3389/fped.2022.980179 (PMC9592840; doi:10.3389/fped.2022.980179)
Supplement: Supplementary file 1 [file Table_1.doc]

Supplementary table 1. Multivariate logistic regression analysis

|  | *P* | *OR* | *95%CI* |
| --- | --- | --- | --- |
| Gestational age | 0.005* | 0.525 | 0.336~0.819 |
| Birth weight | 0.255 | 0.998 | 0.995~1.001 |
| 5-min Apgar score | 0.376 | 0.798 | 0.484~1.315 |
| Surfactant | 0.273 | 1.631 | 0.681~3.908 |
| Intrauterine infection | 0.010* | 3.242 | 1.328~7.913 |
| Invasive mechanical ventilation time | 0.080 | 1.164 | 0.982~1.380 |
| The 3-d Cumulative fluid load | 0.870 | 1.009 | 0.906~1.124 |
| The 7-d Cumulative fluid load | 0.030* | 1.063 | 1.006~1.123 |
| **P*<0.05 was considered statistically significant. | | | |
